# Effects of Replacing Fishmeal with Defatted Black Soldier Fly (*Hermetia illucens* Linnaeus) Larvae Meal in Japanese Eel (*Anguilla japonica*) Diet on Growth Performance, Fillet Texture, Serum Biochemical Parameters, and Intestinal Histomorphology

**DOI:** 10.1155/2022/1866142

**Published:** 2022-12-01

**Authors:** I-Pei Kuo, Ching-Shuo Liu, Shuenn-Der Yang, Shih-Hsiang Liang, Yeh-Fang Hu, Fan-Hua Nan

**Affiliations:** ^1^Freshwater Aquaculture Research Center Chupei Station, Fisheries Research Institute, Council of Agriculture, No. 111, Tai-Ho, Zhubei, Hsinchu 30267, Taiwan; ^2^Department of Aquaculture, National Taiwan Ocean University, No. 2, Beining Road, Keelung 202301, Taiwan; ^3^Animal Industry Division, Livestock Research Institute, Council of Agriculture, No. 112, Farm Road, Hsinhua, Tainan, 71246, Taiwan

## Abstract

An 8-week feeding trial was conducted to investigate the effects of replacing fishmeal with defatted black soldier fly larvae meal (DBSFLM) in the diets of Japanese eel on their growth performance, fillet texture, serum biochemical parameters, and intestinal histomorphology. Six isoproteic (520 g kg^−1^), isolipidic (80 g kg^−1^), and isoenergetic (15 MJ kg^−1^) diets were formulated with fishmeal replacement levels of 0% (R0), 15% (R15), 30% (R30), 45% (R45), 60% (R60), and 75% (R75). The growth performance, feed utilization efficiency, survival rate, serum liver function enzymes, antioxidant ability, and lysozyme activity of fish were not affected (*P* > 0.05) by DBSFLM. However, the crude protein and cohesiveness of the fillet in groups R60 and R75 significantly decreased, and the fillet hardness significantly increased (*P* < 0.05). Additionally, the intestinal villus length significantly decreased in the R75 group, and the goblet cell densities were significantly lower in the R45, R60, and R75 groups (*P* < 0.05). Overall, high levels of DBSFLM did not affect growth performance and serum biochemical parameters but significantly altered fillet proximate composition and texture and intestinal histomorphology (*P* < 0.05). The optimal fishmeal replacement level is 30% with 184 g kg^−1^ DBSFLM.

## 1. Introduction

Fishmeal is the primary source of protein in aquaculture feed, especially for carnivorous fish due to their high dietary protein requirement [1]. However, overfishing and climate change have led to a decrease in wild marine fish [2–4]. To cope with the fishmeal shortage, developing sustainable protein sources for fishmeal replacement is necessary [3, 5].

Insect proteins have similar amino acid profiles to that of fishmeal [6]. Moreover, many insects can transform inedible biowaste or organic side-streams into protein with high conversion efficiency [7]. Therefore, insect proteins, such as the black soldier fly (BSF, *Hermetia illucens* Linnaeus), housefly (*Musca domestica* L.), yellow mealworm (*Tenebrio molitor* L.), and cricket (*Gryllus bimaculatus* De Geer), are regarded as sustainable fishmeal alternatives for aquaculture [8–11]. Among these insects, BSF has received more attention due to its superior feed conversion rate. Moreover, the BSF imagoes only intake water and thus do not disseminate diseases or affect the environmental balance [12, 13].

BSF only feed at the larval stage. Because BSF larvae are self-harvested and substrates are left at the prepupa stage, less labor is required when harvesting. Consequently, BSF larvae are commonly used as animal feed. However, defatting is required to prevent lipid oxidation due to their high content of crude lipids (190–420 g kg^−1^ dry weight; [14, 15]). Replacing dietary fishmeal with defatted BSF larvae meal (DBSFLM) has been studied in various fish. Total fishmeal replacement can be achieved in Atlantic salmon (*Salmo salar* L.) diet by using 148 g kg^−1^ DBSFLM without significant effects on growth performance, apparent digestibility, digestive enzyme activity, or liver histology (*P* > 0.05; [16]). For Japanese seabass (*Lateolabrax japonicus* Cuvier), replacing up to 64% of fishmeal with 192 g kg^−1^ DBSFLM did not alter growth performance or intestinal histology (*P* > 0.05). Moreover, the feed intake significantly increased at a DBSFLM inclusion level of 144 and higher (*P* < 0.05; [11]). Substituting 50% fishmeal with 53 g kg^−1^ DBSFLM in Jian carp (*Cyprinus carpio* var. Jian) diet did not affect growth performance or digestive enzyme activity (*P* > 0.05). However, significantly higher hepatic *hsp70* expression and intestinal histopathological damage have been observed when replacement levels exceeded 75% [17].

Texture is critical for consumer acceptability and market value [18]. A texture profile analysis quantifies textural properties by using a force-time curve through two instrumental compression cycles and is widely used for measuring the texture of meat [19]. Several studies have used a texture profile analysis to evaluate the quality of fish meat. The results have suggested that texture is associated with fishmeal replacement level for European sea bass (*Dicentrarchus labrax* L.), Pengze crucian carp (*Carassius auratus* var. Pengze), Japanese seabass, and Nile tilapia (*Oreochromis niloticus* L.; [20–24]).

Japanese eel is a high-value fish and requires a high level of dietary protein [25]. Blood meal (238 g kg^−1^) can substitute 50% of fishmeal in juvenile Japanese eel (at an initial weight of 6 g). Additionally, the fishmeal replacement level can be elevated to 75% with supplementation of arginine, isoleucine, and methionine [26]. Fishmeal analogue (135 g kg^−1^) can replace fishmeal in the Japanese eel diet at a replacement level of 20% (at an initial weight of 120 g). However, higher replacement levels significantly inhibit growth performance, increase serum liver function enzymes (*P* < 0.05), and lead to intestinal villi impairment [27]. Moreover, the optimal fishmeal replacement level may be associated with the growth stage of Japanese eel. The fishmeal replacement level of fishmeal analogue was decreased to 10% in juvenile Japanese eel (at an initial weight of 9 g) based on growth performance [28].

No study has used DBSFLM as Japanese eel feed. To evaluate the feasibility of replacing fishmeal with DBSFLM in the diet of Japanese eel, fish were fed six isoproteic (520 g kg^−1^ crude protein), isolipidic (80 g kg^−1^ crude lipid), and isoenergetic (15 MJ kg^−1^) diets with fishmeal replacement levels of 0% (R0), 15% (R15), 30% (R30), 45% (R45), 60% (R60), and 75% (R75) for 8 weeks; 0, 91, 184, 275, 367, and 458 g kg^−1^ DBSFLM was administered, respectively. The growth performance, fillet texture, serum biochemical parameters, and intestinal histomorphology of Japanese eel were analyzed.

## 2. Materials and Methods

### 2.1. Experimental Diets

The DBSFLM was provided by a livestock research institute in Xihu Township, Miaoli County, Taiwan. The culture temperature and photoperiod were set at 27 ± 1°C and a 16 : 8 L/D cycle, respectively. Soybean milk residue was used as the rearing diet. The BSF larvae were collected during the sixth instar stage and rinsed with distilled water, which was followed by sterilization in boiling water for 15 s. Sterilized larvae were dried at 80°C for 48 h and subsequently press-defatted using a cold-press oil machine (SunTop, Shenzhen, China). The defatted larvae were pulverized and stored at −20°C until use. The proximate and amino acid composition of fishmeal and DBSFLM are shown in Table 1.

Six experimental diets were each formulated to be isoproteic, isolipidic, and isoenergetic with approximately 520 g kg^−1^ crude protein, 80 g kg^−1^ crude lipid, and 15 MJ kg^−1^ gross energy, respectively. Fishmeal (550 g kg^−1^) and soy protein (205 g kg^−1^) were used as the main sources of protein in the basal diet (R0). The fishmeal was substituted with DBSFLM at levels of 15%, 30%, 45%, 60%, and 75% (R15, R30, R45, R60, and R75, respectively). Diet ingredients were mixed, pelleted, and dried overnight at 50°C. The experimental diets were stored at 4°C until further use. The formulation of the experimental diets is provided in Table 2. The proximate and amino acid composition of experimental diets are shown in Table 3.

### 2.2. Feeding Trial

The Japanese eels (90.74 ± 0.92 g) were randomly divided into six groups in triplicates and placed into 100 L tanks with a recirculating system at a density of 15 fish per tank. To maintain water quality, the recirculating system was equipped with a mechanical and biological filter, thermoregulator, and aerator. The temperature was 26 ± 2°C, and the dissolved oxygen level was above 6 mg L^−1^ during the experiment. Ten percent of water was exchanged daily, and the photoperiod was set at a 12 : 12 L/D cycle. Before the feeding trial, fish were fed the R0 diet for 1 week. Then, fish were fed each experimental diet up to apparent satiation at 09 : 00 and 16 : 00 each day for 8 weeks.

All procedures and investigations involving animals were approved by the Institutional Animal Care and Use Committee of the Freshwater Aquaculture Research Center at the Fisheries Research Institute (approval number 110010).

### 2.3. Sampling

Fish were fasted for 24 h prior to sampling at the end of the feeding trial. All surviving fish were counted and weighed. To perform serum, fillet, and intestinal sampling, five fish from each replicate were randomly selected and anesthetized with 0.5 mL L^−1^ 2-phenoxyethanol (Hayashi Pure Chemical, Osaka, Japan). The blood was collected from the caudal vein using a syringe with a 25 G needle and centrifuged at 3,000 rpm for 10 min. The serum was stored at −80°C for further analysis. After blood collection, the fish were sacrificed and dissected. The viscera and liver were weighed, and the midgut was cut into 2 cm pieces for tissue fixation. The dissected fish were then filleted by hand. The right fillet was homogenized for proximate composition analysis, and the left fillet was used for texture profile analysis.

The weight gain rate (WGR), specific growth rate (SGR), feed intake (FI), feed conversion ratio (FCR), protein efficiency ratio (PER), condition factor (CF), viscerosomatic index (VSI), hepatosomatic index (HSI), and survival rate (SR) were calculated as follows:

(1) $$\begin{array}{c} \text{WGR}\left(\%\right)=\frac{\text{final body weight}\left(\text{g}\right)-\text{initial body weight}\left(\text{g}\right)}{\text{initial body weight}\left(\text{g}\right)}\times 100\%, \end{array}$$

(2) $$\begin{array}{c} \text{SGR}\left(\%\text{da}{\text{y}}^{-1}\right)=\frac{\ln\text{ final body weight}\left(\text{g}\right)-\ln\text{ initial body weight}\left(\text{g}\right)}{\text{days}}\times 100\%, \end{array}$$

(3) $$\begin{array}{c} \text{FI}\left(\text{g }{\text{fish}}^{-1}{\text{day}}^{-1}\right)=\frac{\text{total amount of feed consumed}\left(\text{g}\right)/\left[\left(\text{final number of fish}+\text{initial number of fish}\right)/2\right]}{\text{days}}, \end{array}$$

(4) $$\begin{array}{c} \text{FCR}=\frac{\text{diet intake}\left(\text{g}\right)}{\text{final body weight}\left(\text{g}\right)-\text{initial body weight}\left(\text{g}\right)}, \end{array}$$

(5) $$\begin{array}{c} \text{PER}=\frac{\text{final body weight}\left(\text{g}\right)-\text{initial body weight}\left(\text{g}\right)}{\text{diet intake}\left(\text{g}\right)\times \text{crude protein in diet}\left(\%\right)}, \end{array}$$

(6) $$\begin{array}{c} \text{CF}\left(\text{g c}{\text{m}}^{-3}\right)=\frac{\text{body weight}\left(\text{g}\right)}{\text{total }{\text{length}}^{3}\left(\text{cm}\right)}, \end{array}$$

(7) $$\begin{array}{c} \text{VSI}\left(\%\right)=\frac{\text{visceral weight}\left(\text{g}\right)}{\text{body weight}\left(\text{g}\right)}\times 100\%, \end{array}$$

(8) $$\begin{array}{c} \text{HSI}\left(\%\right)=\frac{\text{hepatopancreas weight}\left(\text{g}\right)}{\text{body weight}\left(\text{g}\right)}\times 100\%, \end{array}$$

(9) $$\begin{array}{c} \text{SR}\left(\%\right)=\frac{\text{number of final fish}}{\text{number of initial fish}}\times 100\%. \end{array}$$

### 2.4. Texture Profile Analysis

The middle section (2.5 cm posterior to the urogenital opening) of fillet was cut for texture profile analysis. A double compression test was performed on the middle section of the fillet using a CT3 texture analyzer (AMETEK Brookfield, Middleboro, MA, USA) with a 4 mm diameter cylinder probe. The test parameters were a compression speed of 1 mm s^−1^, a compression distance of 3 mm, and a trigger load of 10 g. Texture profiles were calculated using the software that accompanied the instrument. The formulations were as follows:

(10) $$\begin{array}{c} \text{Hardness}\left(\text{kg}\right)=\text{maximum force value in the first compression}, \end{array}$$

(11) $$\begin{array}{c} \text{Resilience}=\text{posterior peak area in first compression divided by the anterior peak area in first compression}, \end{array}$$

(12) $$\begin{array}{c} \text{Cohesiveness}=\text{peak area in second compression curve divided by the peak area in first compression curve}, \end{array}$$

(13) $$\begin{array}{c} \text{Springiness}=\text{sample height after first compression divided by the original sample height}, \end{array}$$

(14) $$\begin{array}{c} \text{Gumminess}\left(\text{kg}\right)=\text{cohesiveness}\times \text{hardness}, \end{array}$$

(15) $$\begin{array}{c} \text{Chewiness}\left(\text{MJ}\right)=\text{gumminess}\times \text{springiness}. \end{array}$$

### 2.5. Chemical Composition Analyses

Moisture, crude protein, ash, and crude fiber were analyzed according to the standard methods [29], and crude lipid extraction was conducted according to the method of Folch et al. [30]. Moisture was determined by drying the samples at 105°C until a constant weight was measured. Crude protein (*N* × 6.25) was examined using the Kjeldahl method. Crude lipid was extracted using a chloroform–methanol solvent (2 : 1 *v*/*v*), and the solvent was removed using a vacuum concentrator (EYELA, Tokyo, Japan). Ash was measured through incineration in a muffle furnace (Barnstead Thermolyne, Moorhead, MN, USA) at 550°C for 6 h. Crude fiber was measured with a fiber analyzer (ANKOM Technology, Macedon, NY, USA) after digestion with 1.25% sulfuric acid and 1.25% sodium hydroxide solutions. Nitrogen-free extract (NFE) was calculated as 100% − (moisture + crude protein + crude lipid + ash + crude fiber). Gross energy was calculated as 16.7, 37.4, and 16.7 kJ g^−1^ for crude protein, crude lipid, and NFE, respectively [31]. The amino acid composition was determined using a high-speed amino acid analyzer (HITACHI, Tokyo, Japan) after hydrolyzation in a 4 N methane sulfonic acid at 110°C for 24 h.

### 2.6. Serum Biochemical Parameters

Alanine aminotransferase (ALT) activity was measured using ALT K752 assay kits (BioVision, Milpitas, CA, USA). ALT catalyzed alanine and *α*-ketoglutarate to pyruvate and glutamate. The pyruvate was detected using a probe at 570 nm, and ALT activity was calculated using a pyruvate standard curve. Aspartate aminotransferase (AST) activity was measured using AST K753 assay kits (BioVision, Milpitas, CA, USA). AST catalyzed aspartate and *α*-ketoglutarate to oxaloacetate and glutamate. The glutamate was detected using a probe at 450 nm, and AST activity was calculated using a glutamate standard curve. Total antioxidant capacity (TAC) was measured using TAC K274 assay kits with a Trolox standard (BioVision, Milpitas, CA, USA). Cu^2+^ was reduced to Cu^+^ by both small molecules and proteins. The reduced Cu^+^ was chelated using a probe and measured at 570 nm. For evaluating superoxide dismutase (SOD) activity, an SOD 706002 assay kit (Cayman Chemical, Ann Arbor, MI, USA) was used. SOD catalyzed the dismutation of the superoxide anion to molecular oxygen and hydrogen peroxide. Superoxide anion generated by xanthine oxidase and hypoxanthine reduced tetrazolium to formazan. To investigate SOD activity, the formazan level was measured at 450 nm, and bovine erythrocyte SOD was used as a standard. Lysozyme activity was analyzed using a lysozyme LY0100 (Sigma-Aldrich, St. Louis, MO, USA) assay kit. Lysozyme broke down bacterial cell walls through hydrolysis of the beta-1,4-glycosidic bond in peptidoglycan. To measure lysozyme activity, *Micrococcus lysodeikticus* was used as the substrate, and the lysis reaction was monitored using a decrease in absorbance at 450 nm. The total serum protein concentration was analyzed using protein assay dye reagent concentrate 500-006 (Bio-Rad, Hercules, CA, USA) with bovine serum albumin standard (Sigma-Aldrich, St. Louis, MO, USA) based on the Bradford method. Serum was added to the protein assay dye and incubated for 10 min. Absorbance was measured at 595 nm.

### 2.7. Intestinal Histomorphology

The midguts were fixed in Bouin solution (Sigma-Aldrich, St. Louis, MO, USA) for 24 h. Subsequently, the samples were dehydrated in ethanol, infiltrated in xylene, and embedded in molten paraplast (Sigma-Aldrich, St. Louis, MO, USA). The samples were cut (6 *μ*m) using a rotary microtome (Leica Biosystems, Wetzlar, Germany) and stained with hematoxylin and eosin (Merck, Darmstadt, Germany). The examination was conducted using a light microscope (ZEISS, Oberkochen, Germany) with a charge-coupled device (Microtech, Wheeling, IL, USA). Ten villi from each tissue slice were randomly collected for intestinal histomorphological measurements, including the intestinal diameter, muscular thickness, villus length and width, and goblet cell density, using ImageJ version 1.8.0 (National Institutes of Health, Bethesda, MD, USA).

### 2.8. Statistical Analysis

All data are presented as mean ± standard deviation. Arcsine square root transformation was performed prior to the percentage data analysis. A one-way analysis of variance and Tukey's test were used to test the significance of differences. Differences between means were considered significant when *P* < 0.05. Statistical analyses were conducted using Minitab software version 18.1 (Minitab, State College, PA, USA).

## 3. Results

### 3.1. Growth Performance and Somatic Indices

The growth performance and somatic indices are displayed in Table 4. All experimental diets were fed to the eels daily with no observed rejection. After 8 weeks of the feeding trial, the SR of all treatments was higher than 95% and not significantly different between groups (*P* > 0.05). No significant differences (*P* > 0.05) were observed in the growth performance parameters (final body weight (FBW), WGR, and SGR) between all dietary treatments. Moreover, the feed utilization parameters (FI, FCR, and PER) were not affected (*P* > 0.05) by dietary treatments.

In the case of somatic indices, no significant differences (*P* > 0.05) were observed in the CF and HSI. Although the VSI of fish fed in groups R45 and R75 was significantly lower (*P* < 0.05) than those fed in R15, no significant differences (*P* > 0.05) were observed between all fishmeal-replaced groups and R0.

### 3.2. Fillet Texture and Proximate Composition

The results of the fillet texture profile and proximate composition analyses are presented in Table 5. An increased dietary DBSFLM led to an increase in fillet hardness but a decrease in cohesiveness. Both the fillet hardness and cohesiveness of groups R60 and R75 were significantly different from those of group R0 (*P* < 0.05). By contrast, the other texture parameters (i.e., resilience, springiness, gumminess, and chewiness) were not altered when DBSFLM was included (*P* > 0.05).

No significant differences (*P* > 0.05) were observed in the moisture, crude lipid, and ash content of the fillet between all dietary groups. By contrast, the crude protein content decreased significantly (*P* < 0.05) in the R60 and R75 groups compared with the R0 group, which was consistent with the texture profile results.

### 3.3. Serum Biochemical Parameters

The results related to serum biochemical parameters are shown in Table 6. The differences in ALT, AST, TAC, SOD, and lysozyme activity were nonsignificant (*P* > 0.05) between all dietary groups. By contrast, the total protein (g dL^−1^) of R30 (4.59 ± 0.13) and R75 (4.47 ± 0.03) was significantly lower (*P* < 0.05) than that of R0 (5.04 ± 0.25).

### 3.4. Intestinal Histomorphology

**Figure 1:**
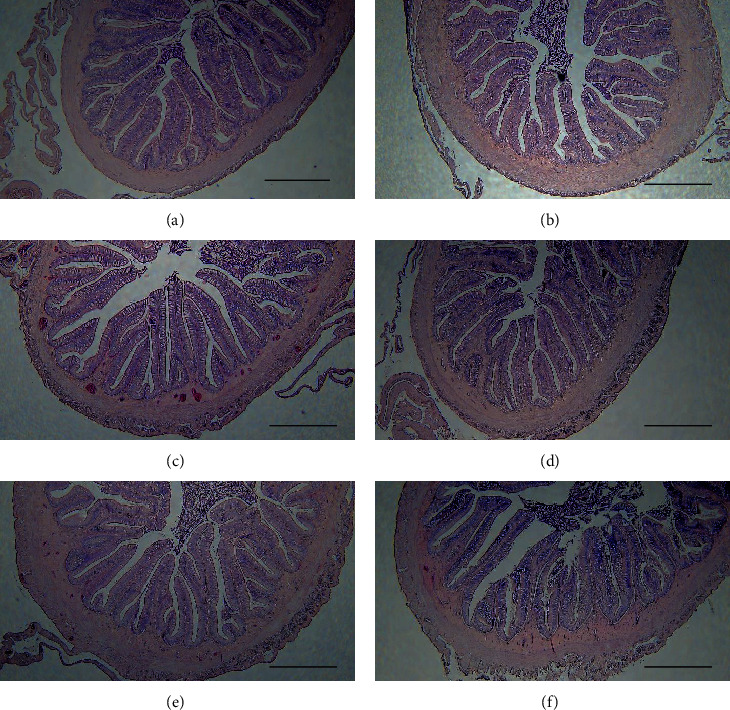
Histological sections of midgut of Japanese eel fed with different replacement levels of defatted black soldier fly larvae meal (DBSFLM): R0 (a), R15 (b), R30 (c), R45 (d), R60 (e), and R75 (f). Midgut sections (6 *μ*m) were stained with hematoxylin and eosin. Bars in bottom right-hand corner represent 500 *μ*m.

**Figure 2:**
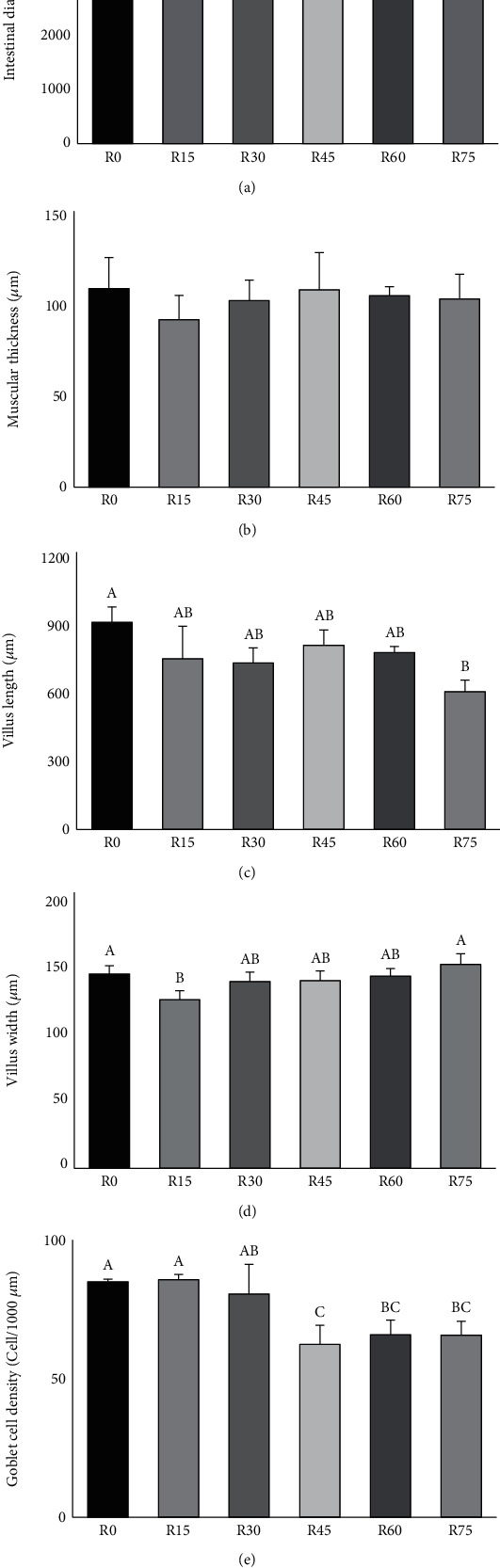
Intestinal diameter (a), muscular thickness (b), villus length (c), villus width (d), and goblet cell density (e) of midgut in Japanese eel fed with different replacement levels of defatted black soldier fly larvae meal (DBSFLM). Data are presented as mean ± standard deviation (*n* = 15). Different superscript letters indicate statistically different values (*P* < 0.05).

The histological sections of the midgut are illustrated in Figure 1, and the measurements of intestinal morphology are presented in Figure 2. The differences in intestinal diameter and muscular thickness were nonsignificant (*P* > 0.05) between all dietary groups. Similar results were observed in the villus width except for group R15, which was significantly lower (*P* < 0.05) than that of groups R0 and R75. However, the villus length and the goblet cell density decreased when the level of fishmeal replacement increased. The villus length of group R75 and the goblet cell densities of groups R45, R60, and R75 were significantly lower (*P* < 0.05) than those of group R0.

## 4. Discussion

### 4.1. Growth Performance and Somatic Indices

BSF is a sustainable protein source and is considered a possible alternative to fishmeal [7]. However, the optimal substitution levels vary among aquaculture species. For instance, complete fishmeal replacement with BSF larvae meal does not affect the growth performance of Atlantic salmon, Nile tilapia, and Jian carp [16, 32, 33]. By contrast, only low dietary levels of fishmeal (20%–28%) can be replaced with BSF larvae meal without inhibiting growth in yellow catfish (*Pelteobagrus fulvidraco* Richardson), white shrimp (*Litopenaeus vannamei* Boone), and barramundi [34–36]. Therefore, studies on the optimal level of BSF larvae meal in different aquaculture species are necessary. To our knowledge, this is the first study to investigate the replacement of fishmeal with DBSFLM for Japanese eel.

Palatability is a critical factor for fishmeal replacement [37]. Several studies have reported that replacing fishmeal with BSF larvae meal reduced diet palatability, resulting in poorer FI and growth performance [38–40]. Low palatability may be caused by oil oxidation due to the richness of the monounsaturated fatty acids in insect meal. Furthermore, the quality of the fatty acid composition of insects is low as it pertains to fish nutritional requirements. Therefore, completing defatting and ensuring a proper drying temperature are advised for producing DBSFLM [41].

In the present study, DBSFLM was defatted using a cold-press oil machine and dried at 80°C for 48 h. No palatability problems or growth suppression were observed when DBSFLM replacement was at or below 75% (458 g kg^−1^ supplementation). Similar results were reported in Jian carp, Nile tilapia, European sea bass, and rainbow trout [17, 32, 42, 43]. DBSFLM (96 g kg^−1^) improved feed palatability and significantly increased FI in Japanese seabass at fishmeal replacement levels higher than 32%, which indicated that DBSFLM may have a higher attractiveness than fishmeal to Japanese seabass [11].

The somatic indices in the present study indicated that the VSI decreased when DBSFLM replacement increased. A similar result was observed in Jian carp with a replacement diet of BSF larvae oil instead of soybean oil; the VSI decreased significantly at replacement levels of 75% and 100% (*P* < 0.05; 19 and 25 g kg^−1^ BSF larvae oil was added, respectively), but the CF and HSI were not altered by dietary treatments (*P* > 0.05; [44]). Therefore, replacing dietary fishmeal with DBSFLM may affect the visceral fat deposition in Japanese eel at 45% replacement and higher. However, further studies are required to fully evaluate the effect of DBSFLM on the visceral fat of Japanese eel.

### 4.2. Fillet Texture and Proximate Composition

Texture profiling using a double compression test is the standard method of textural description and analysis [45]. Moreover, the middle section of Japanese eel fillets was measured in this study because the middle section is the most representative section of fish fillets [46]. In this study, the texture profile results demonstrated that the hardness of Japanese eel fillets significantly increased and the cohesiveness significantly decreased at fishmeal replacement levels of 60% and higher. This finding is consistent with a study by Hu et al. [47] on rice field eel (*Monopterus albus* Zuiew). In that study, a DBSFLM diet replaced fishmeal at levels of 0%, 6.25%, 12.50%, and 18.75% for 10 weeks (0, 53, 105, and 158 g kg^−1^ DBSFLM were added, respectively), and significantly higher muscle hardness and significantly lower cohesiveness (*P* < 0.05) were observed among all DBSFLM dietary groups.

Optimal dietary amino acid levels may differ for growth and fillet quality [48]. Therefore, a texture profile analysis may be useful for evaluating the optimal fishmeal replacement level without adversely affecting fillet texture. Through the use of housefly (*Musca domestica* L.) maggot meal as a fishmeal alternative in Nile tilapia diet, the muscle hardness significantly increased at replacement levels of 25% and higher (110 g kg^−1^ maggot meal; [23]). By replacing 15%, 30%, 45%, and 60% of fishmeal with hydrolyzed feather meal (at 20, 40, 60, and 80 g kg^−1^, respectively), the hardness, springiness, cohesiveness, gumminess, chewiness, and resilience of Pengze crucian carp significantly increased when the replacement level increased (*P* < 0.05). Moreover, the results of a whole-body composition analysis indicated that crude protein levels significantly decreased and crude lipid levels significantly increased at replacement levels of 30% and higher (*P* < 0.05; [24]). Fillet texture can be manipulated by altering the dietary amino acid profile. Taurine supplementation (20 g kg^−1^) in plant protein-based diets (50% fishmeal replacement) significantly increased the hardness and chewiness of European sea bass but significantly decreased the adhesiveness (*P* < 0.05; [49]). Replacing 40%, 50%, and 60% of fishmeal with plant protein significantly decreased the hardness, chewiness, and springiness of turbot (*Scophthalmus maximus* L.). Additionally, 6 g kg^−1^ hydroxyproline supplementation in plant protein-based diets improved the texture, which had similar textures to control (*P* > 0.05; [50]). In this study, the texture of Japanese eel may be altered by dietary amino acid composition at fishmeal replacement levels of 60% and higher.

This study demonstrated that the crude protein content of Japanese eel fillets significantly decreased at fishmeal replacement levels of 60% and higher, which is consistent with the texture profile results. Lin et al. [51] reported that significant changes in muscle myofibrillar, sarcoplasmic, stromal, and crude protein contents were correlated with a crispy texture of grass carp fillets when grass carp were fed broad beans (*Vicia faba* L.). The correlation between fillet texture and crude protein content is still unclear among DBSFLM dietary groups; further studies on muscle protein characteristics are recommended.

### 4.3. Serum Biochemical Parameters

Replacing fishmeal may cause adverse effects on the health of fish, such as liver and intestinal inflammation, antioxidant system imbalance, and immunosuppression [52–54]. In this study, replacing fishmeal with DBSFLM at a replacement level of up to 75% in the diet of Japanese eel did not affect serum liver function enzymes (ALT and AST), antioxidant ability (SOD and TAC), and immune system parameter (lysozyme). Serum protein can primarily be divided into albumin and globulins [55]. Albumin plays a key role in maintaining osmotic balance, transporting substances, and scavenging free radicals [56]. Globulins include hundreds of serum proteins, such as carrier proteins, enzymes, complement proteins, and immunoglobulins [55]. A decrease in total serum protein may reflect a decrease in protein synthesis or an increase in protein loss [57]. Although total serum protein slightly decreased in the R30 and R75 groups, the levels among all groups ranged from 4.47 to 5.04 g dL^−1^ and were regarded as normal values, according to the values of Damusaru et al. [27]. Overall, our results demonstrated that using DBSFLM to replace up to 75% of fishmeal in the Japanese eel diet did not cause negative effects on health-related serum biochemical parameters, which is consistent with previous studies on Atlantic salmon, Jian carp, European sea bass, and African catfish diets [16, 17, 38, 42].

### 4.4. Intestinal Histomorphology

The intestinal histomorphology reflects the nutritional status of fish and can be used to evaluate the potential negative effects of fishmeal alternatives [58]. In particular, villus length and width are related to the surface area of the villi, which is associated with nutrient absorption [59, 60]. Substituting fishmeal completely with 264 g kg^−1^ DBSFLM significantly decreased the villus length of rainbow trout (*P* < 0.05; [61]). Similar results were reported in the diet of grass carp; replacing up to 75% of soybean meal with 190 g kg^−1^ DBSFLM significantly decreased the length of intestinal villus [62].

Goblet cells secrete mucins that are high-molecular-weight glycoproteins and the main structural component of intestinal mucus. The mucus layer formed over the epithelial surface protects the mucosal epithelium against physical and chemical damage but allows for the transport of nutrients. Moreover, goblet cells are involved in the antigen transfer from luminal to lamina propria immune cells [63, 64]. Replacing 50% fishmeal with full-fat BSF larvae meal significantly reduced (*P* < 0.01) the intestinal goblet cell abundance, supranuclear vacuoles, and mucosal fold length in Siberian sturgeon (*Acipenser baerii* Brandt; [65]). Similarly, significant declines in villus width, microvillus height, and goblet cell density were observed in barramundi that were fed with a complete fishmeal replacement diet that mixed poultry by-product meal, tuna hydrolysate, BSF meal, and lupin kernel meal as a fishmeal alternative [60].

Consistent with the aforementioned studies, the villus length of the R75 group and the goblet cell densities of groups R45, R60, and R75 were significantly lower (*P* < 0.05) than those of group R0. The lower villus length and goblet cell densities indicated that DBSFLM exerts adverse effects on the intestinal health of Japanese eel at high levels of fishmeal replacement. However, replacing up to 50% of dietary fishmeal with 400 g kg^−1^ DBSFLM in the rainbow trout diet and up to 64% (DBSFLM inclusion 192 g kg^−1^) in the Japanese seabass diet did not alter their intestinal histomorphology [11, 66].

Chitin is a polysaccharide that is essential for insect exoskeletons, and its role in fish diets is controversial. Although moderate dietary inclusion of chitin exerts positive effects on fish immunity, excess dietary inclusion may suppress the immune system, reduce growth rates, and impair intestinal health [9, 67]. When 10%, 20%, and 30% of fishmeal was substituted with BSF meal (70, 140, and 210 g kg^−1^, respectively) in pearl gentian grouper (*Epinephelus fuscoguttatus* × *E*. *lanceolatus*) diet, the growth performance and intestinal mucosal fold height and muscularis thickness significantly decreased, and the chitinase activity in the distal intestine significantly increased when the dietary BSF meal level increased (*P* < 0.05; [68]). Chitooligosaccharide (GluNAc)_n_ elicits an acute inflammatory cytokine response in human intestinal epithelial-like (Caco-2) cells [69]. The chitinase of Japanese eel has been identified in its stomach, and N-acetylglucosamine (GluNAc) and (GluNAc)_2_ are the final products of chitin hydrolysate [70]. Although 100 g kg^−1^ chitin did not inhibit the growth performance of Japanese eel, the effect of chitin on intestinal health has not been studied [71]. The chitin contents of partially and highly DBSFLM were 50 and 69 g kg^−1^, respectively [72]. In this study, the highest DBSFLM inclusion (R75) was 458 g kg^−1^. Therefore, the calculated chitin inclusion was in the range of 22.6 to 31.6 g kg^−1^ in the R75 diet. To clarify the influence of chitin on intestinal histomorphology, suppling BSF-derived chitin in Japanese eel diet is recommended in future studies.

## 5. Conclusion

This study demonstrated that replacing up to 75% of dietary fishmeal with DBSFLM did not cause adverse effects on the growth performance and serum biochemical parameters of Japanese eel diet. However, the histomorphology demonstrated adverse effects on intestinal health at fishmeal replacement levels of 45% and higher, and the crude protein content and texture of the fillet were significantly altered at fishmeal replacement levels of 60% and higher (*P* < 0.05). Thus, 30% of fishmeal can be replaced with 184 g kg^−1^ DBSFLM in Japanese eel without adverse effects on growth performance, fillet texture, serum biochemical parameters, and intestinal histomorphology.

## Figures and Tables

**Table 1:** Proximate and amino acid composition of fishmeal and DBSFLM.

	Fishmeal	DBSFLM
Proximate composition (g kg^−1^)		
Moisture	63.0	28.2
Crude protein	667.0	600.5
Crude lipid	106.0	81.2
Ash	136.0	81.1

*Amino acid (g kg^−1^ of crude protein)*		
Essential amino acid		
Arginine	61.7	40.3
Histidine	26.2	24.7
Isoleucine	44.3	33.8
Leucine	75.9	57.9
Lysine	84.2	50.3
Methionine	25.1	5.1
Phenylalanine	42.8	35.3
Threonine	40.5	30.6
Tryptophan	8.6	2.8
Valine	52.8	50.1

Nonessential amino acid		
Alanine	65.7	53.6
Aspartic acid	98.8	76.7
Cystine	8.3	4.5
Glutamic acid	137.0	90.1
Glycine	69.4	46.2
Proline	44.1	42.8
Serine	38.7	32.8
Tyrosine	36.5	58.0

Abbreviation: DBSFLM: defatted black soldier fly larvae meal.

**Table 2:** Formulation of experimental diets.

Ingredients (g kg^−1^)	R0^†^	R15	R30	R45	R60	R75
Fishmeal	550	468	395	303	220	138
Soy protein	205	205	205	205	205	205
DBSFLM	0	91	184	275	367	458
Squid liver meal	30	30	30	30	30	30
Fish oil	34	35	36	38	39	40
Vitamin premix^‡^	20	20	20	20	20	20
Vitamin C^§^	5	5	5	5	5	5
Mineral premix^¶^	20	20	20	20	20	20
*α*-Starch	60	54	48	42	36	30
CMC	40	40	40	40	40	40
Cellulose	36	32	27	22	18	14
Total	1000	1000	1000	1000	1000	1000

Abbreviation: DBSFLM: defatted black soldier fly larvae meal; CMC: sodium carboxymethyl cellulose. ^†^R0–R75 mean percentage of fishmeal replacement. ^‡^Vitamin premix (kg^−1^) contains retinol 1 MIU, calciferol 0.2 MIU, *α*-tocopherol 12.5 IU, menadione sodium bisulfite 1.25 g, thiamin 2.75 g, riboflavin 4 g, pyridoxine 3.25 g, cyanocobalamin 2 mg, nicotinic acid 13.5 g, calcium pantothenate 7.7 g, biotin 125 mg, folic acid 750 mg, ascorbic acid-2-phosphate 20 g, and inositol 22.5 g. ^§^Feed-grade, 50% ascorbic acid-2-phosphate. ^¶^Mineral premix (kg^−1^) contains calcium 80 g, phosphate 100 g, potassium 15 g, magnesium 15 g, copper 5 g, iodine 5 mg, iron 1 g, manganese 0.8 g, selenium 50 mg, zinc 7.5 g, and cobalt 5 mg.

**Table 3:** Proximate and amino acid composition of experimental diets.

	R0	R15	R30	R45	R60	R75
Proximate composition (g kg^−1^)						
Moisture	26.1	26.5	20.9	25.3	21.1	24.0
Crude protein	524.9	525.2	527.3	523.2	522.0	520.7
Crude lipid	83.6	83.3	83.7	84.0	83.4	87.3
Ash	128.1	125.4	118.5	113.9	108.4	102.9
Crude fiber	11.8	34.3	41.9	54.5	60.9	32.3
NFE	225.5	205.3	207.7	199.1	204.2	232.8
Gross energy (MJ kg^−1^)	15.7	15.3	15.4	15.2	15.3	15.9

*Amino acid (g kg^−1^ crude protein)*						
Essential amino acid						
Arginine	61.3	62.8	59.6	56.3	55.1	57.3
Histidine	22.8	24.9	25.1	25.6	27.0	28.7
Isoleucine	43.2	44.6	42.9	41.1	40.6	41.8
Leucine	78.8	80.8	77.3	73.4	72.5	73.5
Lysine	72.9	73.1	68.8	65.1	62.6	62.6
Methionine	17.3	18.5	17.8	14.8	13.9	13.0
Phenylalanine	42.9	44.3	42.9	41.0	41.0	42.4
Threonine	38.1	38.3	36.6	35.5	34.6	35.0
Tryptophan	1.1	1.2	1.0	1.5	1.7	1.6
Valine	49.7	52.5	51.9	51.7	52.5	54.7

Nonessential amino acid						
Alanine	59.2	61.0	57.8	56.9	56.2	59.5
Aspartic acid	94.8	96.1	92.9	89.8	89.4	92.7
Cystine	7.3	7.3	6.5	6.2	6.3	6.3
Glutamic acid	147.2	146.3	138.6	131.2	126.3	133.6
Glycine	53.2	54.1	53.4	53.2	53.3	55.6
Proline	41.0	44.1	44.2	45.2	47.4	49.9
Serine	40.5	41.8	40.7	39.3	39.9	41.4
Tyrosine	35.8	41.1	43.0	46.4	50.8	55.1

Abbreviation: NFE: nitrogen-free extract.

**Table 4:** Growth performance and somatic indices of Japanese eel fed with different replacement levels of DBSFLM.

	R0	R15	R30	R45	R60	R75
IBW (g)	90.11 ± 3.66	91.71 ± 3.26	89.38 ± 1.10	91.36 ± 1.91	90.4 ± 1.38	91.49 ± 1.78
FBW (g)	166.15 ± 2.36	165.71 ± 9.01	173.13 ± 7.87	171.17 ± 1.83	172.37 ± 7.43	170.72 ± 9.19
WGR (%)	84.64 ± 9.86	80.7 ± 7.57	93.76 ± 10.08	87.45 ± 5.96	90.66 ± 6.99	86.52 ± 6.70
SGR (% day^−1^)	0.87 ± 0.08	0.84 ± 0.06	0.94 ± 0.07	0.90 ± 0.05	0.92 ± 0.05	0.89 ± 0.05
FI (g fish^−1^ day^−1^)	2.03 ± 0.03	1.94 ± 0.06	1.92 ± 0.06	1.94 ± 0.04	1.98 ± 0.04	2.02 ± 0.02
FCR	1.55 ± 0.08	1.51 ± 0.06	1.34 ± 0.06	1.39 ± 0.13	1.40 ± 0.06	1.47 ± 0.10
PER	1.23 ± 0.06	1.26 ± 0.05	1.42 ± 0.06	1.38 ± 0.13	1.37 ± 0.06	1.31 ± 0.09
SR (%)	95.56 ± 3.85	97.78 ± 3.85	95.56 ± 3.85	97.78 ± 3.85	95.56 ± 3.85	95.56 ± 3.85
CF (g cm^−3^)	0.15 ± 0.01	0.15 ± 0.00	0.15 ± 0.01	0.15 ± 0.01	0.16 ± 0.01	0.16 ± 0.01
VSI (%)	4.17 ± 0.06^ab^	4.42 ± 0.39^a^	3.98 ± 0.02^ab^	3.65 ± 0.46^b^	3.79 ± 0.09^ab^	3.53 ± 0.13^b^
HSI (%)	1.22 ± 0.06	1.30 ± 0.04	1.29 ± 0.10	1.19 ± 0.04	1.18 ± 0.06	1.19 ± 0.07

Abbreviation: DBSFLM: defatted black soldier fly larvae meal; IBW: initial body weight; FBW: final body weight; WGR: weight gain rate; SGR: specific growth rate; FI: feed intake; FCR: feed conversion ratio; PER: protein efficiency ratio; SR: survival rate; CF: condition factor; VSI: viscerosomatic index; HSI: hepatosomatic index. Data are presented as the mean ± standard deviation (*n* = 3 for growth performance and *n* = 15 for somatic indices). Different superscript letters in the same row indicate statistically different values (*P* < 0.05).

**Table 5:** Fillet texture profile and proximate composition of Japanese eel fillet fed with different replacement levels of DBSFLM.

	R0	R15	R30	R45	R60	R75
Texture profile						
Hardness (kg)	2.66 ± 0.45^c^	3.02 ± 0.16^bc^	3.22 ± 0.23^abc^	3.31 ± 0.34^abc^	3.93 ± 0.39^a^	3.53 ± 0.12^ab^
Resilience	0.40 ± 0.05	0.38 ± 0.04	0.33 ± 0.03	0.31 ± 0.03	0.26 ± 0.04	0.30 ± 0.03
Cohesiveness	0.42 ± 0.10^a^	0.41 ± 0.05^a^	0.34 ± 0.01^ab^	0.32 ± 0.01^ab^	0.26 ± 0.03^b^	0.26 ± 0.03^b^
Springiness	0.60 ± 0.08	0.73 ± 0.04	0.73 ± 0.04	0.67 ± 0.06	0.70 ± 0.05	0.68 ± 0.04
Gumminess (kg)	1.09 ± 0.26	1.21 ± 0.15	1.06 ± 0.06	1.01 ± 0.08	1.03 ± 0.11	0.91 ± 0.13
Chewiness (MJ)	19.98 ± 7.28	32.60 ± 5.20	22.61 ± 2.39	20.34 ± 2.32	21.09 ± 1.59	20.72 ± 6.50

Proximate composition (g kg^−1^)						
Moisture	647.6 ± 1.9	647.4 ± 6.3	648.4 ± 7.9	630.4 ± 5.6	630.6 ± 8.3	632.3 ± 8.2
Crude protein	165.3 ± 2.1^a^	162.9 ± 1.2^ab^	165.0 ± 3.4^a^	160.6 ± 2.4^ab^	156.4 ± 2.4^b^	157.2 ± 3.4^b^
Crude lipid	184.8 ± 3.9	180.2 ± 13.0	187.8 ± 3.8	207.2 ± 5.6	205.1 ± 10.3	200.7 ± 18.5
Ash	12.0 ± 0.3	12.0 ± 0.3	12.1 ± 0.5	12.3 ± 0.5	11.6 ± 0.5	12.3 ± 0.3

Abbreviation: DBSFLM: defatted black soldier fly larvae meal. Data are presented as the mean ± standard deviation (*n* = 15). Different superscript letters in the same row indicate statistically different values (*P* < 0.05).

**Table 6:** Serum biochemical parameters of Japanese eel fed with different replacement levels of DBSFLM.

	R0	R15	R30	R45	R60	R75
ALT (mU mL^−1^)	1.02 ± 0.03	0.89 ± 0.06	0.96 ± 0.17	0.84 ± 0.05	1.09 ± 0.21	1.02 ± 0.09
AST (mU mL^−1^)	0.93 ± 0.03	1.06 ± 0.37	0.96 ± 0.17	0.63 ± 0.11	0.95 ± 0.30	0.87 ± 0.21
TAC (nmol *μ*L^−1^)	32.5 ± 0.35	31.38 ± 0.65	30.96 ± 0.86	32.93 ± 1.06	31.42 ± 0.74	31.57 ± 1.01
SOD (U mL^−1^)	147.87 ± 13.16	150.99 ± 14.90	136.42 ± 4.12	137.71 ± 11.3	156.69 ± 11.76	171.39 ± 29.76
Lysozyme (U mL^−1^)	154.81 ± 30.11	141.3 ± 49.83	176.97 ± 33.54	168.8 ± 41.7	189.44 ± 26.36	160.71 ± 6.79
Total protein (g dL^−1^)	5.04 ± 0.25^a^	5.01 ± 0.15^a^	4.59 ± 0.13^bc^	4.99 ± 0.10^ab^	4.73 ± 0.15^abc^	4.47 ± 0.03^c^

Abbreviation: DBSFLM: defatted black soldier fly larvae meal; ALT: alanine aminotransferase; AST: aspartate transaminase; TAC: total antioxidant capacity; SOD: superoxide dismutase. Data are presented as the mean ± standard deviation (*n* = 15). Different superscript letters in the same row indicate statistically different values (*P* < 0.05).

## Data Availability

The data that support the findings of this study are available from the corresponding author upon reasonable request.
